# More than just a histone deacetylase: cytoplasmic SIRT6 facilitates fatty acid oxidation through ACSL5 deacetylation: SIRT6-mediated ACSL5 deacetylation regulating FFA metabolism

**DOI:** 10.3724/abbs.2023030

**Published:** 2023-03-06

**Authors:** Shuoshuo Li, Zengqiang Yuan

**Affiliations:** The Brain Science Center Beijing Institute of Basic Medical Sciences Beijing 100850 China

Nonalcoholic fatty liver disease (NAFLD) is the most common liver disease worldwide; it affects up to 25%–30% of the global population. NAFLD encompasses a spectrum of conditions, ranging from hepatic steatosis and nonalcoholic steatohepatitis (NASH) to fibrosis and cirrhosis. Further progression of NAFLD can lead to hepatocellular carcinoma [1]. Despite being researched for decades, effective therapeutic drugs for NAFLD remain lacking. The pathogenesis of NAFLD is complex, and its underlying mechanisms remain elusive. Free fatty acids (FFAs) are crucial for life and essential for cellular functions in mammals; however, excessive FFAs contribute to the pathogenesis of NAFLD. A recent study published by Hou *et al*. [2] suggested that cytoplasmic SIRT6 is involved in NAFLD progression by regulating FFA metabolism.

## SIRT6 Translocates to the Cytoplasm in Response to Palmitic Acid Treatment

Elevated levels of FFAs, especially saturated FFAs (SFAs), are closely associated with obesity and contribute to the pathogenesis of metabolic syndromes, including NAFLD and cardiovascular disease. Palmitic acid (PA, C16:0) is a saturated long-chain fatty acid (FA) that is among the most abundant dietary and plasma FFAs. Excessive PA overload in the liver induces lipotoxicity, leading to the development of NAFLD [3]. However, the molecular mechanisms involved in sensing FFAs and modulating lipotoxicity are not completely understood. SIRT1, the most studied sirtuin, is a nutrient sensor and metabolic modulator that regulates hepatic lipid metabolism through the induction of FGF21 [4]. In a recent issue of *Molecular Cell*, Hou *et al*. [2] found that SIRT6 directly binds with PA; this binding stimulates SIRT6 to interact with the exporter protein that facilitates SIRT6 translocation, which is a prerequisite for binding with its substrate in the cytoplasm. In contrast, PA efficiently stimulates SIRT6 activity to promote substrate deacetylation.

**Figure 1:**
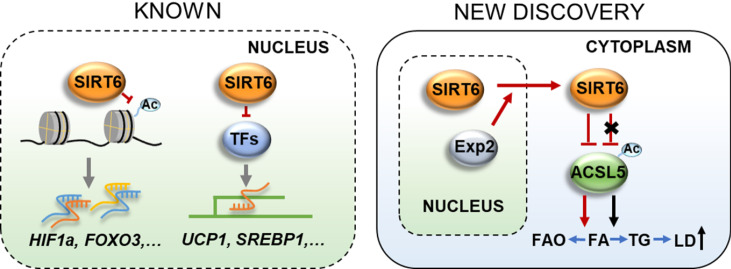
Figure 1 Cytoplasmic SIRT6 facilitates fatty acid oxidation As previously reported, SIRT6 is predominantly located in the nucleus and preferentially associated with gene transcriptional regulation, Hou et al. [2] found that SFAs-induced SIRT6 cytoplasmic localization is mediated by Exp2. Cytoplasmic SIRT6 facilitates fatty acid oxidation through deacetylation of ACSL5, thereby ameliorating high-fat diet-induced NAFLD development.

SIRT6 is predominantly located in the nucleus and is preferentially associated with chromatin [5]. It also plays critical roles in metabolism, oxidative stress, and DNA repair [6]. As a master metabolic regulator, SIRT6 is essential for maintaining glucose homeostasis and lipid metabolism at multiple levels: first, through histone deacetylation activity, SIRT6 corepresses the transcription of key metabolism factors by HIF1α and FoxO3 to direct glucose flux towards glycolysis and reduce LDL-cholesterol levels, respectively; second, SIRT6 interacts with or modifies important transcription factors, such as FoxO1 and SREBP1, to regulate hepatic glucose, lipid, and cholesterol regulatory genes [7] ( Figure 1). However, these SIRT6-regulated metabolic changes are based on its effect on metabolism-related gene expression due to SIRT6 nuclear localization. SIRT6 is also present in the cytoplasm [8]. However, the role of cytoplasmic SIRT6 in the regulation of FFA metabolism remains unclear. Hou *et al*. [2] demonstrated that the cytoplasmic localization of SIRT6 is mediated by Exp2, which is known to mediate importin α export from the nucleus. Importantly, this accumulation is induced by SFAs, specifically PA. They provided the first evidence that SIRT6 modulates lipid metabolic homeostasis in the cytoplasm by directly interacting with and deacetylating a nonhistone substrate.

## SIRT6-Mediated ACSL5 Deacetylation Promotes Lipid Expenditure through Fatty Aid Oxidation

Acetylation plays a major role in metabolic regulation and modulates enzymes involved in gluconeogenesis and FA metabolism. Emerging evidence indicates that protein acetylation is a key regulatory mechanism in NAFLD development. Hence, it is constructive to identify acetylated regulators of FA metabolism that can control NAFLD development. Hou *et al*. [2] found that the deacetylation of ACSL5, a key enzyme in FA activation, contributes to NAFLD progression. A previous study has shown that ACSL5 is also involved in fatty acid oxidation (FAO), triglyceride (TG) synthesis, acylceramide generation, and cardiolipin level maintenance [9]. Hou *et al*. [2] demonstrated that cytoplasmic SIRT6 interacts with ACSL5 and promotes the deacetylation of ACSL5 at K98, K361, and K367, thereby maintaining ACSL5 activity and channeling FFA metabolism. In addition, they showed that hepatic overexpression of either ACSL5-WT or deacetylated ACSL5-3KR mimic, notably, the protective function of ACSL5 that is missing in hepatic-specific *SIRT6*-KO mice, emphasizing the necessity of deacetylation by SIRT6 for ACSL5 activity.

The pathogenesis of NAFLD is complex, and the metabolic dysregulation of FFAs is central to this process. An imbalance in FFA supply (uptake or *de novo* lipogenesis) and disposal (mainly FAO or very low-density lipoprotein secretion) in the liver leads to elevated levels of hepatic FFAs, which causes lipotoxicity and promotes the development of NAFLD. The two major fates of FFAs in hepatocytes are FAO and TG formation; however, PA, one of the most abundant FFAs, is poorly incorporated into TG [3]. Therefore, FAO largely contributes to the disposal of excess hepatic PA, and increasing FAO protects against NAFLD. This hypothesis was supported by a study by Hou *et al*. [2]. Their data demonstrated that ACSL5 deacetylation by SIRT6 can protect against lipotoxicity and maintain FFA metabolism homeostasis in an NAFLD model by enhancing FAO. Despite this, increased FAO, especially incomplete FAO, may lead to the overproduction of reactive oxygen species (ROS), which induces oxidative stress, impairs mitochondrial function, and contributes to the progression of NAFLD [10]. Therefore, the protective mechanism underlying ROS clearance requires further investigation.

## Perturbation of the SIRT6–ACSL5 Axis Contributes to the Progression from Simple Steatosis to Steatohepatosis

During simple steatosis, a highly reversible NAFLD stage, multiple cellular stress responses are activated to accelerate FFA disposal, thereby preventing the aggravation of NAFLD. Previous reports indicated that SIRT6 loses some of its ability to regulate the gene expression of critical metabolic enzymes owing to its decreased expression level. Hepatic SIRT6 deficiency leads to fatty liver in mice, and the expression of SIRT6 is reduced in human NAFLD samples [6]. Interestingly, Hou *et al*. [2] found that the cytoplasmic SIRT6–ACSL5 axis serves as a protective mechanism to counter NAFLD pathological changes, preventing the progression from simple steatosis to NASH under metabolic stress by facilitating FAO and enhancing mitochondrial adaptation. While simple steatosis alone is relatively benign, NASH greatly increases the risk of advanced liver diseases, such as cirrhosis and hepatocellular carcinoma. Many protective adaptations are abolished at this stage. In mice and patients with simple steatosis that exhibit slightly lower levels of SIRT6, most ACSL5 proteins are deacetylated by cytoplasmic SIRT6. However, in mice with severe NASH or patients who exhibit extremely low levels of SIRT6, little SIRT6 can be translocated. Therefore, a significant increase in ACSL5 acetylation was observed, indicating that the SIRT6–ACSL5 axis-mediated protective role may be absent in NASH. Thus, SIRT6-mediated ACSL5 deacetylation represents a new regulatory pathway in FA metabolism, and targeting this pathway may be a promising approach for NAFLD/NASH prevention and treatment.
